# In Situ Experimental Analysis and Performance Evaluation of Airport Precast Concrete Pavement System Subjected to Environmental and Moving Airplane Loads

**DOI:** 10.3390/ma17215316

**Published:** 2024-10-31

**Authors:** Yoo Bong Kim, Seong-Min Kim

**Affiliations:** Department of Civil Engineering, Kyung Hee University, Yongin 17104, Gyeonggi, Republic of Korea; kiddy72@khu.ac.kr

**Keywords:** airplane load, airport precast concrete pavement, curling, dynamic load, environmental load, fatigue failure, taxiway

## Abstract

The behavior of airport precast concrete pavement (APCP) involving new design and construction concepts was experimentally analyzed under environmental and moving airplane loads, and the long-term performance of the APCP was evaluated using fatigue failure analysis. The strain characteristics and curling behavior of the APCP under environmental loads were comprehensively analyzed. The APCP slabs exhibited a pronounced curling phenomenon similar to conventional concrete pavement slabs. The dynamic response of the APCP subjected to impact loads was analyzed by performing heavy weight deflectometer tests. The test results confirmed that the vertical deformation of the APCP was small and within the typical range of vertical deformation of conventional concrete pavement. The dynamic strain response of the APCP under moving airplane loads was then analyzed and the strain variation during day and night times was compared. The strains during the day were found to be significantly larger than those at night under airplane loads because of the curling phenomenon of the APCP slabs. Finally, the long-term performance of the APCP was evaluated using fatigue failure analysis based on the obtained behavior. Even using the most conservative fatigue failure prediction model, the service life of the APCP was ascertained to be more than 30 years. Based on the overall results of this study, it is concluded that the APCP, which is designed to reduce slab thickness by placing reinforcing bars in the slabs via reinforced concrete structural design, exhibits typical behavior of concrete pavements and can be successfully applied to airport pavement rehabilitation.

## 1. Introduction

For airport runways and taxiways, Portland cement concrete pavements (hereinafter referred to as concrete pavements), which are rigid pavements with excellent durability, are most widely used; asphalt concrete pavements (hereinafter referred to as asphalt pavements), which are flexible pavements, are also used. Airport pavements require maintenance due to deterioration and damage. If there are multiple runways, other runways and taxiways may be used temporarily to allow for the repair of the runway and taxiway pavements. However, at airports with a small number of runways, airplane operations must be restricted at night and pavement repairs must be made rapidly. If cast-in-place concrete pavements are constructed to completely replace the aged pavement at an airport with a large number of airplane operations and an insufficient number of runways, the concrete curing period will cause significant inconvenience to the use of the airport. The airport precast concrete pavement (APCP) method, which improves or repairs the pavements of runways and taxiways using concrete slabs prefabricated in a factory, reduces construction time and improves pavement quality even though the construction cost is high. In this respect, the APCP is a very advantageous method.

Precast concrete pavement (PCP) has been used for roads and airports since 1931, when the hexagon-shaped system was first developed in the Soviet Union [1,2,3]. In the Soviet Union, PCPs were continuously improved and widely used until the mid-1970s [3,4,5,6]. In the United States of America, PCPs were first used in a missile mat at the Ohio River Division Laboratory of the U.S. Army Engineer Division in 1956 [3] and were applied to a highway in South Dakota in 1968 [3,7]. In Europe, PCPs have been used for airport construction since 1947 in countries including France, England, and Belgium [3,8]. Recently, for road pavements, various types of PCPs have been developed and a number of studies have been conducted to improve their performance [9,10,11,12,13,14,15,16,17,18,19,20,21,22,23,24,25,26,27]. For airport pavements, most studies of APCPs have been performed to evaluate the performance [28,29,30,31,32,33,34,35,36,37,38]. Some studies on APCPs have been focused on the construction [39,40,41,42,43,44], behavior analysis [45,46,47,48], and materials [49].

The most common PCP construction method involves removing the entire surface layer of the aged existing pavement, repairing the exposed base layer, installing precast slabs, and grouting empty spaces. If the PCP methods used in existing road pavements are applied to airport pavements as they are, the slab weight increases significantly due to the large slab area and thickness, making handling inconvenient and construction difficult. For reference, in Korea, a typical jointed concrete pavement (JCP) slab in road pavements has an area of 6 m by 4 m and a thickness of 0.28 m, whereas a JCP slab in airport pavements has an area of 6 m by 6 m and a thickness of 0.5 m. Additionally, if the surface layer of the existing pavement is removed completely for repair, the base layer must be reconditioned, which takes time and is expensive. In this research, in order to more conveniently convert an aged taxiway pavement composed of asphalt pavement to concrete pavement, the thickness of the concrete slab was reduced and a reinforcement design was performed to enable it to withstand increasing tensile stresses due to the slab thickness reduction. In addition, the APCP was applied more efficiently by eliminating processes such as base layer compaction and base surface preparation by leaving a small portion of the thickness of the existing asphalt pavement layer rather than removing the entire asphalt pavement layer.

The objective of this study is to analyze the behavior of this type of APCP and predict its performance. To this end, during the construction of the APCP, various gauges were installed to measure the behavior due to environmental and moving airplane loads, and the long-term performance of the APCP was predicted based on the analyzed behavior. This paper describes this research in detail.

## 2. APCP Design and Measurement Plan

### 2.1. Design of New Concept APCP

**Figure 1:**
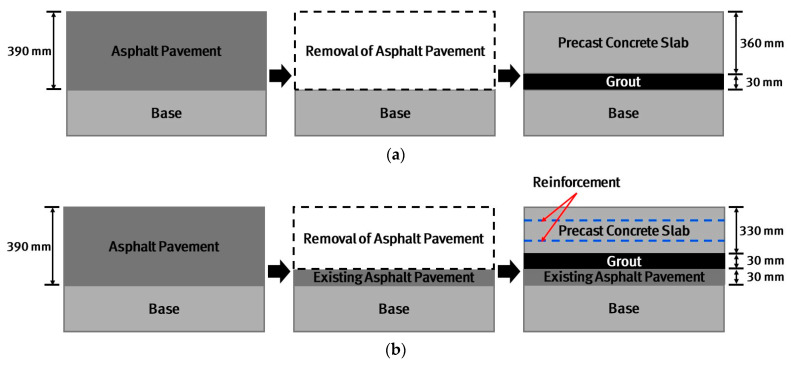
APCP design and construction plan: (**a**) Typical plan; (**b**) Modified plan employed in this study.

In order to convert the existing taxiway asphalt pavements to cement concrete pavements using the precast concrete pavement method, generally, as shown in Figure 1a, all asphalt pavement layers are removed, precast slabs are installed, and the empty spaces between the slab and base are grouted. However, in this case, since the base is disturbed during the process of removing all the asphalt pavement layers, thorough compaction and surface preparation work on the base must be performed to ensure bearing capacity. That is, the construction time and cost increase for base compaction and surface preparation. Therefore, in this study, in order to improve these shortcomings, as shown in Figure 1b, the taxiway asphalt pavements at Gimpo International Airport in Seoul, Korea are removed while leaving 30 mm of thickness; the precast slab thickness is reduced by 30 mm to account for this. As a result, there is no need for base reconditioning work, which helps to reduce construction time, the biggest advantage of the precast concrete pavement method, while also reducing construction costs. The decrease in slab rigidity as the slab thickness decreases is compensated for by placing reinforcing bars in the slab. The traditional jointed concrete pavement design is performed to determine the slab thickness based on the mechanistic analysis of behavior and the empirical analysis of performance. Since reinforcing bars are used in this study, the slab design is performed based on the reinforced concrete slab design. The bending moment of an APCP slab is obtained using finite element analysis and reinforcement design is performed according to the design code [50]. Since the traditional concrete pavement design does not consider the existence of reinforcing bars, slab thickness can be reduced by implementing the reinforced concrete slab design. Reinforcing bars with a diameter of 13 mm are placed at a height of 50 mm from the bottom of the slab and 80 mm from the top of the slab. The spacing of the longitudinal reinforcing bars is designed to be 150 mm, and the spacing of the transverse reinforcing bars is designed to be 300 mm.

**Figure 2:**
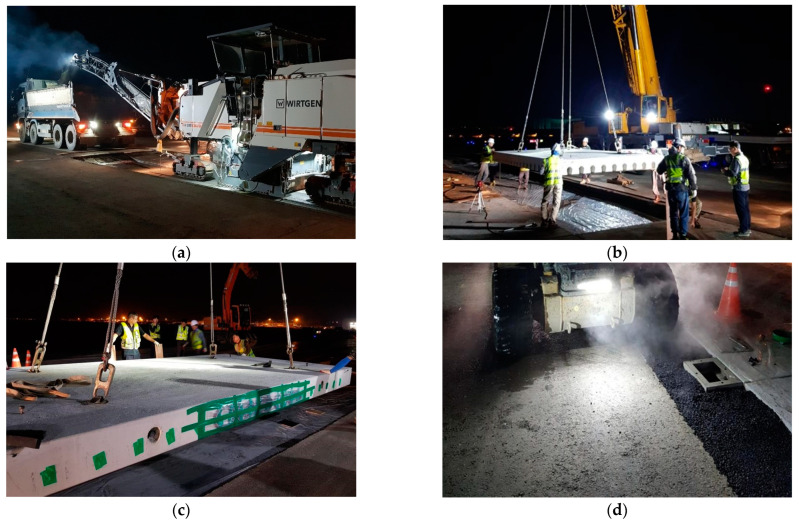
Construction of APCP: (**a**) Removing existing asphalt pavement; (**b**) Lifting precast slabs; (**c**) Installing precast slabs; (**d**) Asphalt paving of surrounding area.

The taxiway APCP section was constructed using a total of 90 precast concrete slabs, with 18 rows of precast slabs installed in the direction of airplane movement and 5 rows in the transverse direction. As shown in Figure 2, the existing asphalt pavement was first removed, then the precast slabs were lifted and placed in the planned locations, and the levels between the slabs were adjusted. Afterwards, the empty space at the bottom of the precast slabs was grouted and the area around the APCP sections were filled with asphalt mixtures to complete the construction. It is noted that since APCP is a type of jointed concrete pavement, dowel bars are used between slabs for load transfer purposes. There are also gaps between the slabs to allow for slab expansion and contraction due to temperature changes.

### 2.2. Installation of Measurement Gauges

**Figure 3:**
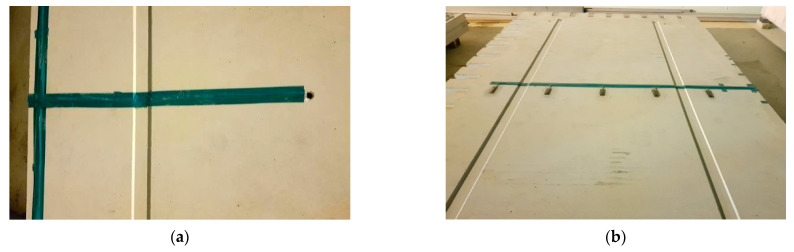

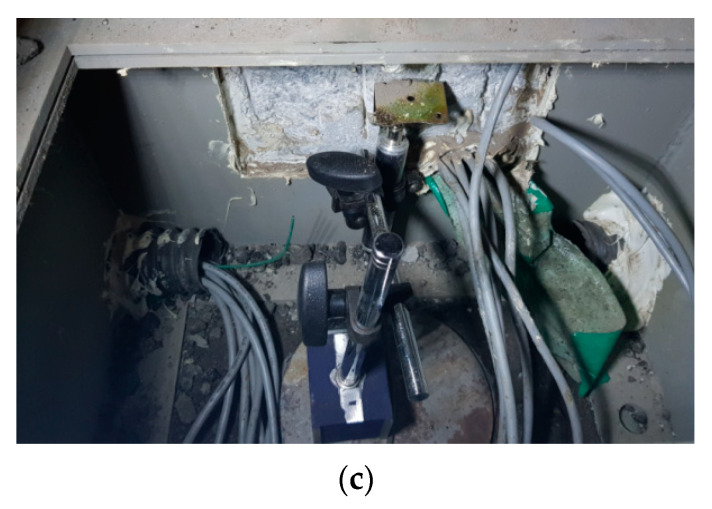
Measurement gauge installation: (**a**) Temperature gauge installation; (**b**) Strain gauge installation; (**c**) Displacement gauge installation.

**Figure 4:**
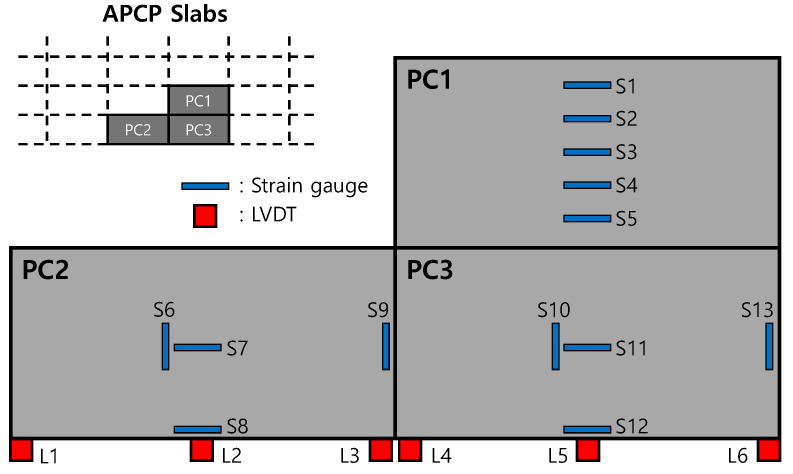
Location and numbering of measurement gauges.

To analyze the behaviors of the APCP, the temperature measurement gauges, strain gauges, and displacement gauges were installed as shown in Figure 3. In order to measure the temperature changes by slab depth, which are environmental loads acting on the APCP, thermocouples, the temperature measurement sensors, were installed at the top, mid-depth, and bottom of two precast slabs as shown in Figure 3a. To measure the strains of the APCP slabs under environmental and moving airplane loads, the strain gauges were installed at the bottom of the precast slabs as shown in Figure 3b. As shown in Figure 4, the strain gauges were installed at a total of 13 locations on three slabs and were installed to measure strains at various parts of the slabs. Gauges of S6, S9, S10, and S13 are for measuring transverse strains, and all other gauges are for measuring longitudinal strains.

To measure the curling behavior, which is the vertical displacement change behavior of the APCP slab due to environmental loads, the linear variable displacement transducers (LVDTs) were installed immediately after the slab installations, as shown in Figure 3c. As shown in Figure 4, the LVDTs were installed at six locations on the sides of two slabs. Gauges of L2 and L5 are for measuring the vertical displacements of the central part of the slab side, and the rest are all for measuring the vertical displacements of the corner of the slab.

## 3. Behavior of APCP Under Environmental Loads

In this study, the temperature transfer characteristics of the APCP were analyzed by measuring the temperature change in the slab due to changes in ambient temperature, and the deformation characteristics of the APCP due to environmental loads were identified by analyzing the measured strains of the slab. In addition, the vertical displacements of the APCP were measured to analyze the curling behavior and the relationship with temperature changes was identified.

### 3.1. Temperature Variation Through Slab Depth

**Figure 5:**
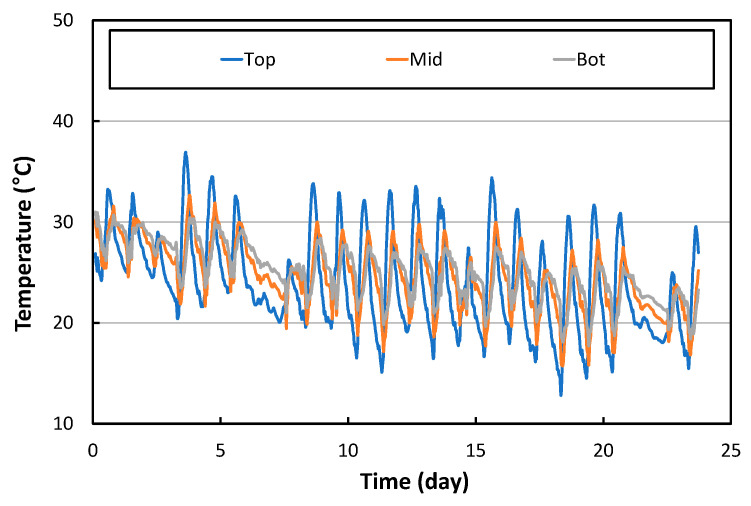
Temperature variation through slab depth.

The temperature changes at the top, middle, and bottom of the APCP slab were measured and shown in Figure 5. The temperature change is largest at the top of the slab, and the temperature change decreases as the depth increases. In addition, it can be seen that the times at which the maximum and minimum temperatures occur are somewhat delayed as the depth of the slab increases. This phenomenon is the same as the temperature change characteristic generally observed in cast-in-place concrete pavements [51,52].

**Figure 6:**
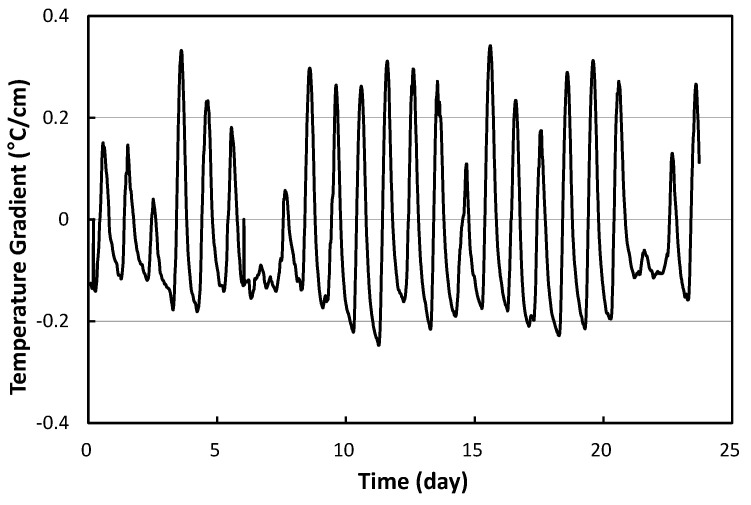
Linear vertical temperature gradient of APCP.

To analyze the curling behavior of the APCP, the vertical temperature gradient of the slab must be obtained using the temperature at each slab depth. In fact, the temperature varies nonlinearly with slab depth. It is possible to obtain a linear vertical temperature gradient that causes a bending curvature that matches the bending curvature of the slab caused by this non-linear vertical temperature gradient [53]. Figure 6 shows the variation in the linear vertical temperature gradient obtained using the temperature measurement data in Figure 5. In the figure, a negative vertical temperature gradient occurs when the temperature at the top of the slab is lower than the temperature at the bottom of the slab, causing curl up of the slab. On the other hand, a positive vertical temperature gradient causes curl down of the slab since the temperature at the top of the slab is higher than that of the bottom of the slab. Also, if the slope of the tangent line of the graph is positive, it means that the slab curls down, and vice versa.

### 3.2. Strains of APCP

**Figure 7:**
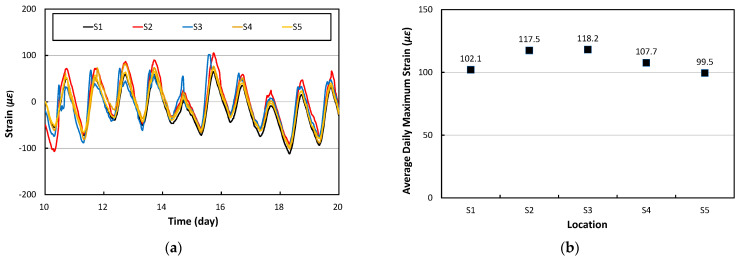
Longitudinal strains of PC1: (**a**) Strain variation; (**b**) Daily maximum strain.

The variations in the longitudinal strains over time measured at the gauges of S1 through S5 installed along the transverse direction of the PC1 slab are shown in Figure 7a. The strains appear to increase during the day and decrease at night. Figure 7b shows the daily maximum longitudinal strain at each gauge location obtained by averaging the difference between the daily maximum and minimum strains measured over 10 days. It can be seen that the daily maximum longitudinal strain is largest at S3, the center of the slab, and the strain decreases somewhat toward the side edges of the slab. The deviation of longitudinal strain according to the lateral position in the center of the slab was found to be approximately 16%.

**Figure 8:**
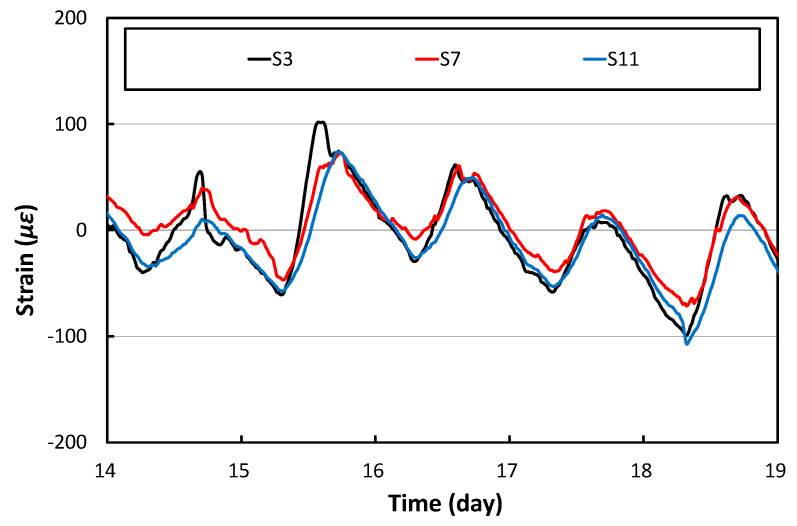
Longitudinal strains at the center of slabs.

Figure 8 shows the longitudinal strains at the center of the three different slabs of PC1, PC2, and PC3. Since all three APCP slabs are longitudinally connected to adjacent slabs, the constraints for longitudinal deformation are similar. Therefore, as can be seen in the figure, the overall strain time histories are similar although there are very slight differences between the slabs.

**Figure 9:**
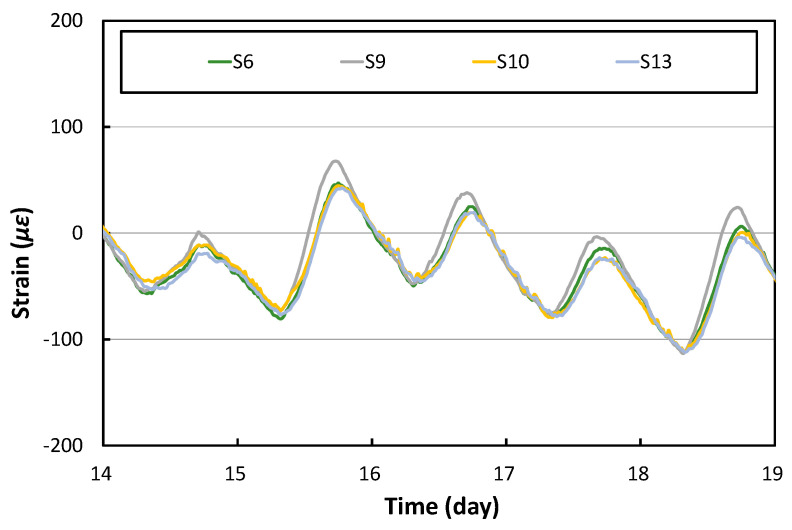
Transverse strains at the center of slabs.

Figure 9 shows the transverse strains of the slabs. The gauge positions of S6 and S10 are the center of the PC2 and PC3 slabs, respectively, and the gauge positions of S9 and S13 are the central portion of the slab joint. As shown in the figure, the transverse strain time histories of the center of the slabs are also very similar both in the center of the slab and at the joint. In addition, when comparing the previously analyzed longitudinal strains with the transverse strains, it can be seen that they are almost similar although the longitudinal strains are very slightly larger.

**Figure 10:**
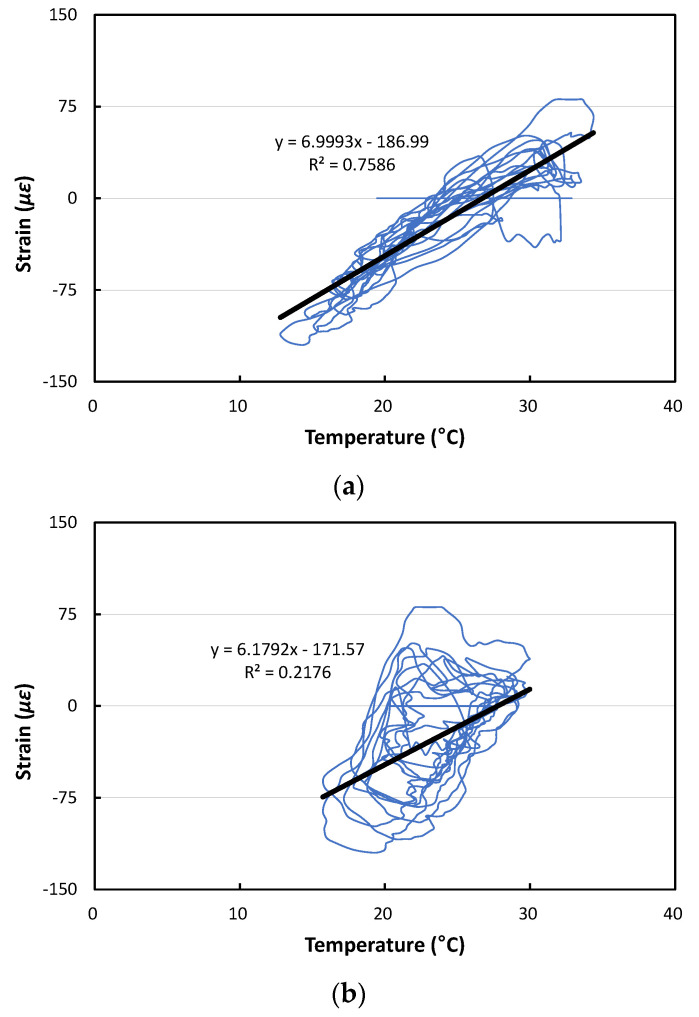

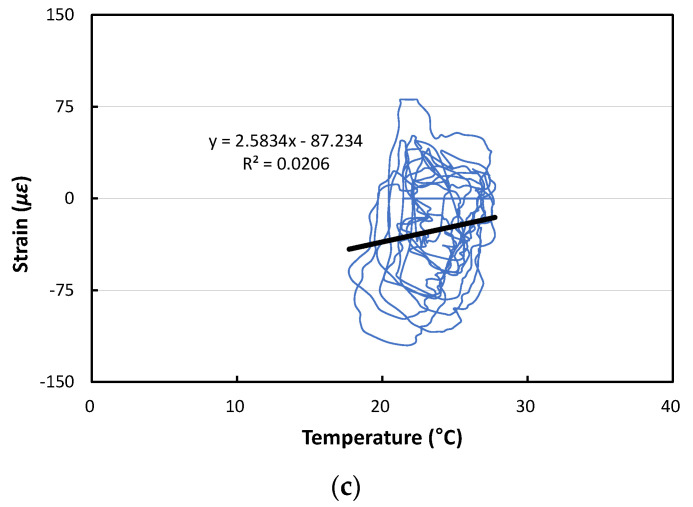
Relationships between strain and temperature: (**a**) Based on temperatures at top of slab; (**b**) Based on temperatures at middle of slab; (**c**) Based on temperatures at bottom of slab.

Figure 10 shows the relationships between temperature and strain, and also shows the linear trend lines. Even though the strain gauges were attached to the bottom of the slab, the strain change according to the temperature change by slab depth appears most linearly when analyzing based on the temperatures at the top of the slab as shown in Figure 10a. It was found that a strain change of about 7 με occurred at the bottom of the slab per unit temperature change at the top of the slab. Therefore, if the temperature at the top of the slab changes by about 50 °C per year, it is predicted that a strain change of about 350 με will occur at the bottom of the slab.

### 3.3. Curling Behavior of APCP

**Figure 11:**
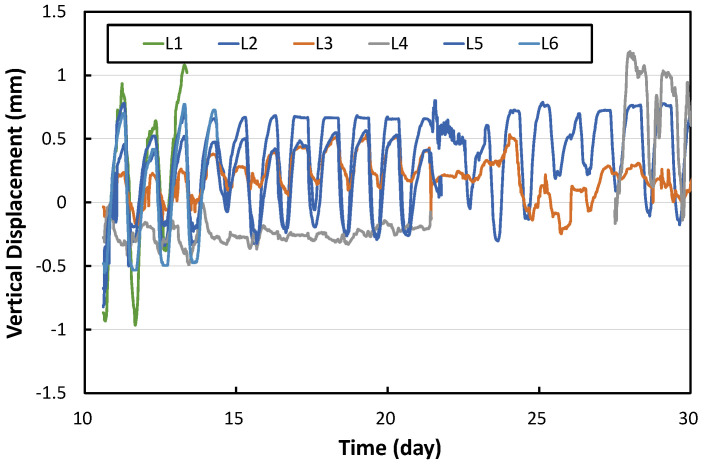
Vertical displacements of slab edge.

In concrete pavements, a temperature difference occurs between the top and bottom of the slab due to environmental loads, which causes curling, a bending phenomenon of the slab. The vertical displacement measurement gauges were installed to measure the curling behavior of the APCP slabs, and the results after eliminating data errors are shown in Figure 11. During the day, the slab curls down, causing the vertical displacement of the slab edge to occur in the negative (downward) direction. Conversely, at night, the slab curls up, causing the vertical displacement of the slab edge to occur in the positive (upward) direction.

**Figure 12:**
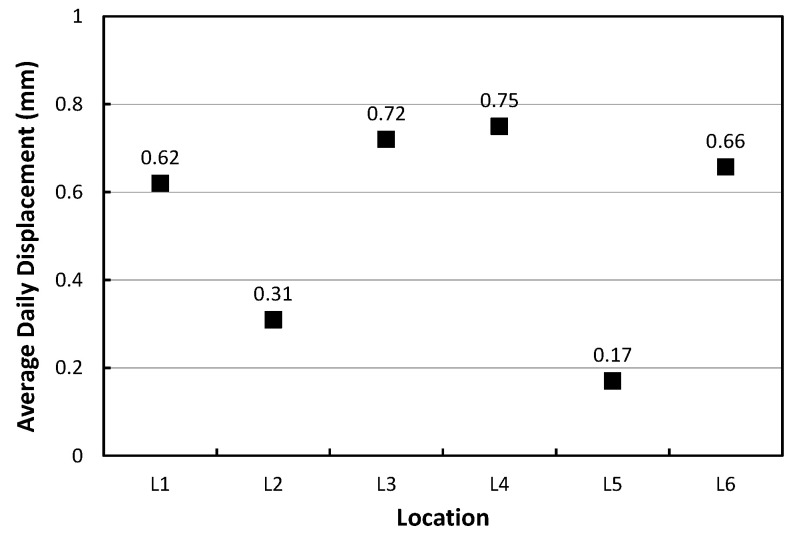
Average daily vertical displacements.

Figure 12 shows the average daily vertical displacement changes in the APCP slabs. The vertical displacements measured using the L1, L3, L4, and L6 gauges installed at the corners of the slabs are clearly larger than the vertical displacements measured using the L2 and L5 gauges installed at the center of the slab edge. This means that the APCP slabs are separated from the supporting layer and exhibit curling behavior like typical concrete pavement slabs.

**Figure 13:**
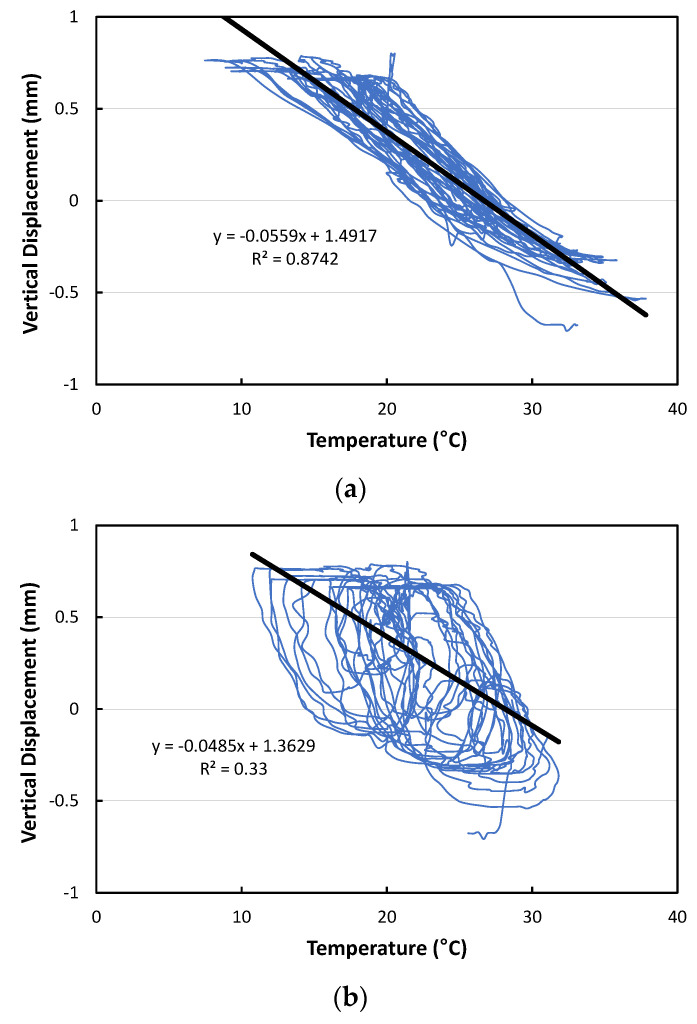

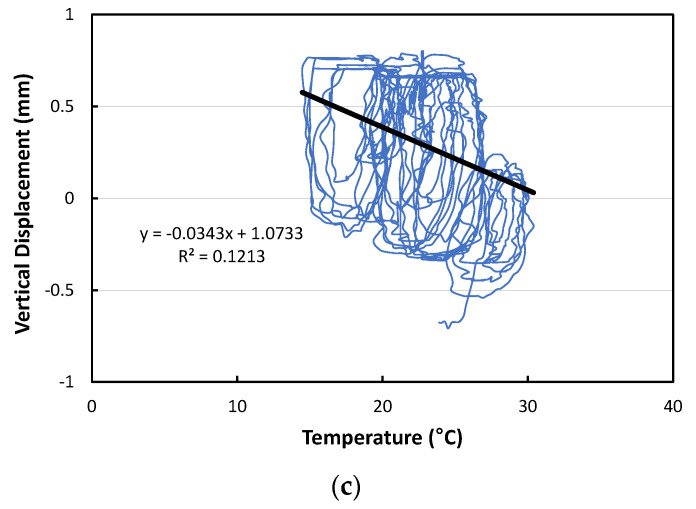
Relationships between curling displacement and temperature: (**a**) Based on temperatures at top of slab; (**b**) Based on temperatures at middle of slab; (**c**) Based on temperatures at bottom of slab.

The relationships between temperature and curling displacement are shown in Figure 13 and the linear trend lines are also shown in the figure. The curling displacement change according to the temperature change by slab depth appears most linearly when analyzing based on the temperatures at the top of the slab as shown in Figure 13a. It was found that the vertical displacement per unit temperature change of about 0.056 mm/°C occurred at the corner of the slab. If the largest daily change in temperature at the top of the slab is assumed to be 20 °C, it is predicted that the change in curling displacement of the slab will be up to 1.12 mm. This is considered to be a similar range compared to the curling displacement in typical concrete pavements, which is approximately 1.2 mm [53].

## 4. Behavior of APCP Under HWD Loads

**Figure 14:**
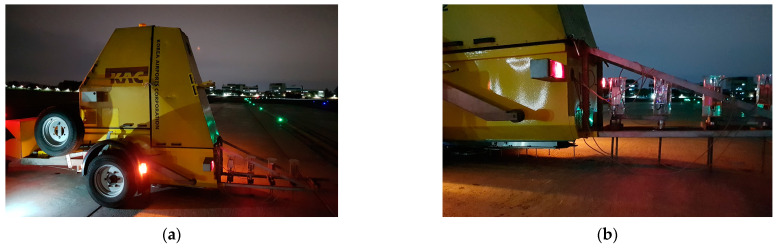
Dynamic load test using HWD: (**a**) HWD equipment; (**b**) Isolated geophones.

**Figure 15:**
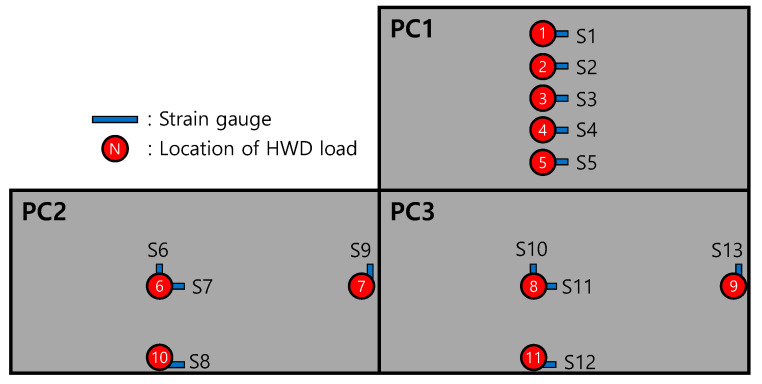
Locations of HWD tests.

Since the gear load of airplane varies significantly depending on the type of airplane, number of passengers, cargo loads, etc., it is difficult to analyze comprehensively the behavior of the APCP according to the magnitude and location of the airplane gear load. Therefore, in order to analyze the behavior of the APCP when a dynamic load of a certain magnitude is applied, a dynamic load test was performed using a heavy weight deflectometer (HWD) shown in Figure 14. Figure 15 shows each HWD loading position where the strain gauges were installed, and the HWD loads were applied at magnitudes of 127 kN and 216 kN.

**Figure 16:**
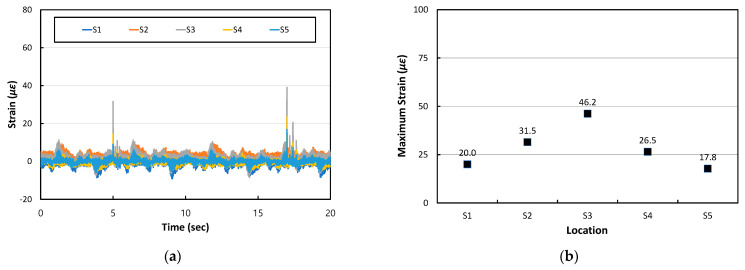
Dynamic strain responses at different transverse locations under HWD load at S3: (**a**) Dynamic strain time histories; (**b**) Maximum strains.

When the HWD load was applied to position S3, the center of the PC1 slab, the dynamic responses and maximum longitudinal strains at different transverse positions S1 to S5 were analyzed and shown in Figure 16. As shown in Figure 16a, the dynamic strain responses increase significantly at the moments when two different magnitudes of the HWD loads are applied. The largest value of the maximum strain occurs at the loaded location of S3 as shown in Figure 16b, and the maximum strain decreases as the distance from the loaded location increases.

**Figure 17:**
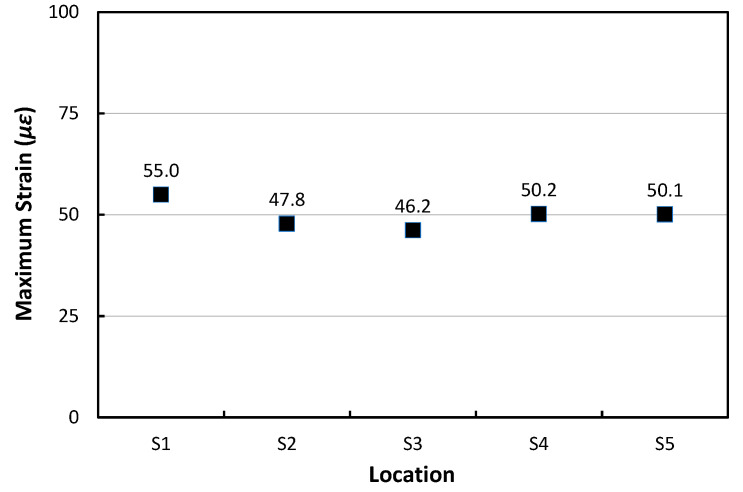
Maximum longitudinal strains at different transverse locations under HWD loads.

When the HWD load was applied to different transverse positions S1 to S5, respectively, the maximum longitudinal strain at each position was analyzed and shown in Figure 17. It can be seen that the maximum strain when the dynamic load acts on the central part of the slab is smaller than when it acts near the edge of the slab. This phenomenon is a typical behavior of concrete pavement slabs and means that the flexural rigidity at the central part of the slab is greater than that near the slab edge.

**Figure 18:**
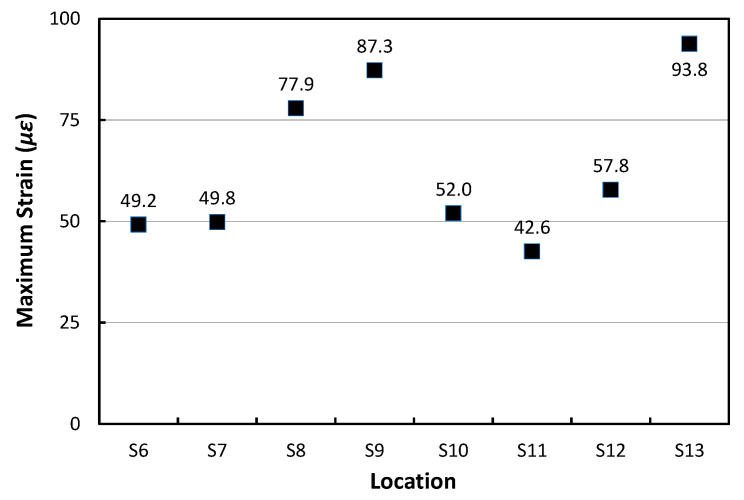
Maximum strains at different locations under HWD loads.

Figure 18 shows the maximum dynamic strain of the APCP slab at each HWD test location from the HWD load number 6 to 11 (corresponding gauges of S6 to S13) when the load is applied at each location. It can be clearly seen that the maximum strains are relatively small when the dynamic loads act on the central part of the slab, the HWD load number 6 and 8 (corresponding gauges of S6, S7, S10, and S11). The maximum strains increase when the dynamic loads are applied near the slab joint and edge, the HWD load number 7, 9, 10, and 11 (corresponding gauges of S8, S9, S12, and S13). In addition, the maximum transverse strains measured at S9 and S13 of the slab joints were found to be the largest, and the maximum longitudinal strains measured at S8 and S12 of the slab edge were found to be relatively smaller.

In order to examine the ratio between the longitudinal and transverse strains of the APCP slab, the strains measured by the gauges of S6 and S7, and S10 and S11, where the longitudinal and transverse strain gauges were installed at the same location were used. As shown in Table 1, the transverse and longitudinal strains obtained from the S6 and S7 gauges have almost the same values, but when comparing the transverse and longitudinal strains obtained from the S10 and S11 gauges, the transverse strain is slightly larger. Therefore, the average ratio of the transverse strain to the longitudinal strain was analyzed to be approximately 1.1.

## 5. Behavior of APCP Under Moving Airplane Loads

To analyze the behavior of the APCP under moving airplane loads, the dynamic strain responses were measured at a frequency of 100 Hz using the S1 to S5 strain gauges installed at the PC1 slab while the airplanes were moving. The strain measurements of the APCP due to moving airplane loads were performed separately during the day and at night. The dynamic strains were measured for a total of 18 airplanes, 12 during the day and 6 at night. Table 2 shows the airplane types and measurement times.

**Figure 19:**
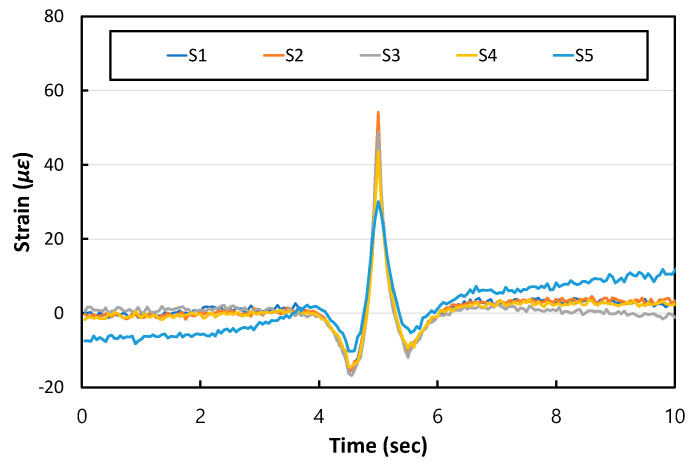
Dynamic strain responses to N1 airplane loads.

Figure 19 shows the dynamic strain responses of the APCP when N1 airplane moves and passes the gauge positions. In all gauges, when the airplane approaches the measurement location, some negative strains occur first, and when the airplane arrives at the measurement location, the maximum positive strains occur. Then, when the airplane passes the measurement location, some negative strains occur again, and then the strains eventually become 0. A similar pattern of the dynamic strain response is observed for all other airplanes. It is noted that the APCP is a type of plate structure on a foundation. Therefore, the APCP behavior under moving airplane loads should be similar to the behavior of other plate structures on foundations. The phenomenon of positive and negative strains occurring when vehicle moves is a typical behavior of a plate structure on a foundation [54], and the APCP behavior obtained in this study also confirmed this phenomenon.

**Figure 20:**
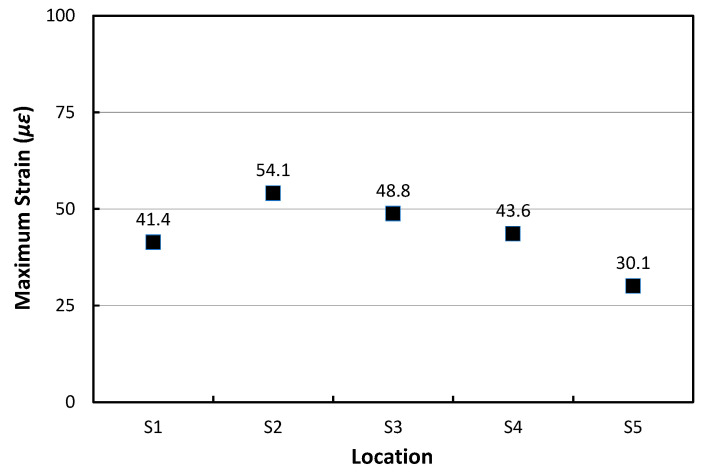
Maximum strains at different transverse locations when N1 airplane moves.

As shown in Figure 20, if the maximum strains obtained from the gauges are compared when N1 airplane moves, the largest maximum strain of 54.1 με occurs at the S2 gauge. As the distance from the S2 gauge in the transverse direction increases, the maximum strain gradually decreases, with the smallest maximum strain occurring at the farthest S5 gauge. This means that the airplane gear load passed closest to the S2 gauge location.

The results of analyzing the maximum strains at the gauge locations for all airplanes considered in the experiments are shown in Table 3. The measurement results for all airplanes also show that the largest maximum strain occurs at a certain gauge location and that the maximum strain decreases as the transverse distance from that gauge increases. Additionally, when comparing the average values of the maximum strains during the day and night times, the maximum strain is larger during the day.

**Figure 21:**
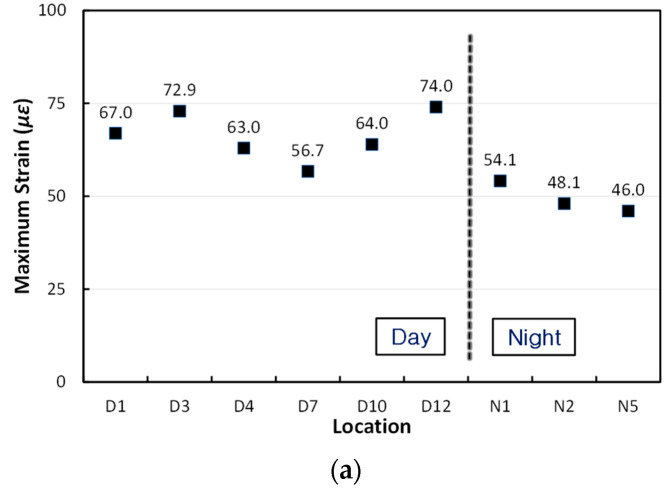

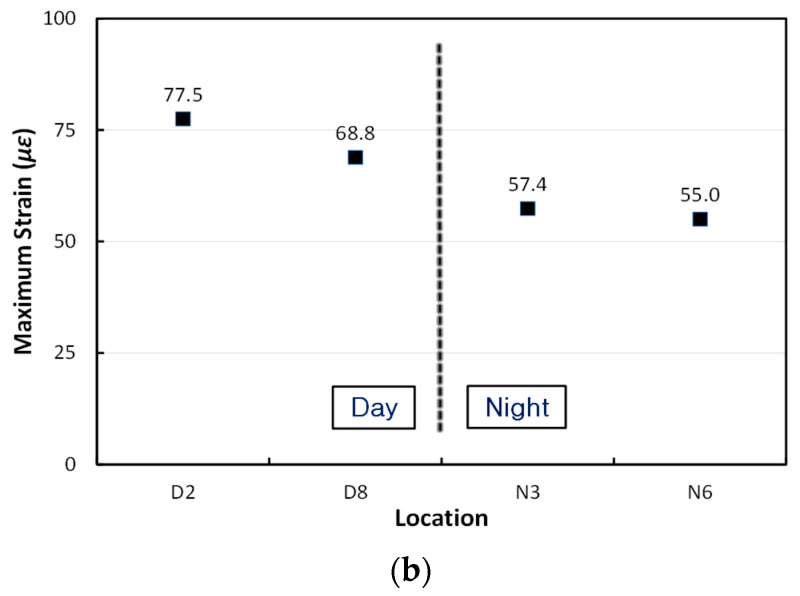
Comparison of maximum strains during day and night times: (**a**) B737 airplanes; (**b**) A320 airplanes.

In order to more specifically compare and analyze the strains of the APCP due to moving airplane loads during the day and night, the analysis was performed considering the airplane types that passed the test section the most, the B737 and A320. The maximum strains of the APCP when the B737 airplanes passed are shown in Figure 21a. During the daytime, the maximum strains occurred in the range of 56.7 με to 74.0 με, and during the night, the maximum strains occurred in the range of 46.0 με to 54.1 με. It can be seen that the largest maximum strain during the night is even smaller than the smallest maximum strain during the day. The average maximum strain during the day is 66.3 με, and at night that is 49.6 με. Figure 21b also shows the maximum strains of the APCP when the A320 airplanes passed. Although the number of data is small, it can be seen that, as in the case of the B737 airplanes, the largest maximum strain during the night is significantly smaller than the smallest maximum strain during the day. The average maximum strain during the day is 73.2 με, and at night that is 56.2 με.

In both the B737 and A320 airplanes, the maximum strains during the day are found to be greater than the maximum strains at night. This is due to the curling phenomenon of the slab. At night, the slab curls up and the airplane load is applied while the central part of the slab is firmly supported by the underlying support layers, so the strains in the central part of the slab appear small. However, during the day, the slab curls down and the airplane load is applied while the central part of the slab is not firmly supported by the underlying support layers, causing the strains in the central part of the slab to become larger. Likewise, changes in the behavior of pavement slabs under wheel loads due to slab curling have also been previously reported in road pavement studies [55].

The vertical deformation of concrete pavements subjected to vehicle loads is very small, typically less than 1 mm, and rutting, a permanent deformation that commonly occurs in asphalt pavements, does not occur in concrete pavements, so the vertical deformation is not considered as a performance index in the concrete pavement design [56,57,58]. An analysis was performed to evaluate the vertical deformation of the APCP under airplane loads. In the HWD tests, the maximum vertical deformation of the APCP was measured as 0.38 mm. Since the tests were conducted at night, the daytime deformation would be about 25% larger, as shown in Table 3. Therefore, the maximum vertical deformation of the APCP was found to be approximately 0.48 mm, which is within the typical range of vertical deformation of concrete pavement. This means that the vertical deformation of the APCP does not affect the serviceability of pavement.

## 6. Performance Evaluation of APCP

Since the tensile stress occurring in the APCP slab is designed to be less than the tensile strength, damage to the slab does not occur due to a one-time load. However, fatigue failure may occur due to repeated loading over a long period of time. The fatigue failure prediction methods applicable to concrete pavement slabs include various formulas such as the S-N curve developed by the American Concrete Institute [59], Darter equation [60], Aas–Jakobsen equation [61], and Roesler equation [62].

The S-N curve is a method of predicting fatigue failure by defining the relationship between the stress level, which is the ratio of stress to strength, and the number of repeated loads at which fatigue failure occurs. In this method, if the tensile stress due to the repetitive load occurring in the slab is less than about 50% of the tensile strength (if the stress level is less than 0.5), fatigue failure does not occur even during the load cycle of 10 million [59]. In this case, there is no need to consider fatigue failure.

The fatigue failure prediction equation according to Darter [60] is shown in Equation (1):

(1) $$Log\left(N_{f}\right)=17.61(1-\frac{\sigma }{MOR})$$

where $N_{f}$ is the number of load application until failure, *σ* is the applied maximum stress, and *MOR* is the modulus of rupture of the concrete.

The fatigue failure prediction equation according to Aas–Jakobsen [61] is shown in Equation (2):

(2) $$\frac{\sigma_{max}}{f_{c}^{\prime }}=1-\beta \left(1-R\right){Log}_{10}N_{f}$$

where $\sigma_{max}$ is the maximum applied stress, $f_{c}^{\prime }$ is the compressive or tensile strength of the concrete, *β* is a coefficient experimentally determined to be 0.064, *R* is the ratio of the minimum stress to the maximum stress, and $N_{f}$ is the number of load application until failure.

The fatigue failure prediction equation according to Roesler [62] is shown in Equation (3):

(3) $$N_{f}={\left(\frac{1.2968}{\left(\frac{\sigma }{{MOR}_{beam}}\right)}\right)}^{32.57}$$

where $N_{f}$ is the number of load applications until failure, *σ* is the applied maximum stress, and ${MOR}_{beam}$ is the modulus of rupture of the concrete beam.

In this study, the tensile stresses of the APCP slabs due to moving airplane loads were analyzed and the long-term performance of the APCP was evaluated using the fatigue failure formulas. The tensile stresses of the APCP slabs due to moving airplane loads can be obtained using Equation (4) by utilizing the measured strains:

(4) $$\sigma_{x}=\frac{E}{1-\nu^{2}}(\varepsilon_{x}+\nu \varepsilon_{y})$$

where $\sigma_{x}$ is the longitudinal stress, *E* is the elastic modulus, *ν* is Poisson’s ratio, $\varepsilon_{x}$ is the longitudinal strain, and $\varepsilon_{y}$ is the transverse strain.

An APCP slab is 6 m long in the longitudinal direction and 3 m long in the transverse direction. Since the longitudinal length of the slab is larger than the transverse length, cracks may occur along the transverse direction caused by the longitudinal tensile stresses when the slab becomes fatigued. Therefore, the long-term performance analysis was performed using the longitudinal tensile stresses.

To determine the longitudinal stress of the APCP slab, not only the longitudinal strain but also the transverse strain is required, as shown in Equation (4). Since only the longitudinal strains under the moving airplane loads were measured, the transverse strain was assumed to be 1.1 times the longitudinal strain using the strain analysis results under the HWD loads shown in Table 1. In addition, the typical values of 29 GPa and 0.15 were used, respectively, for the elastic modulus and Poisson’s ratio of concrete. Since the maximum longitudinal strain was found to be 77.5 με under the D2 airplane loads according to Table 3, this strain was used conservatively in further analyses. The maximum longitudinal stress occurring in the APCP slab can be obtained using Equation (4), and as a result, the maximum stress of 2.68 MPa was acquired.

Table 4 shows the results of the flexural tensile strength test [63] of the APCP slab, and the average flexural tensile strength was found to be 5.77 MPa. Therefore, since the tensile stress of the APCP slab obtained in this experiment was 2.68 MPa, the stress level, which is the ratio of tensile stress to tensile strength, was analyzed to be about 0.46.

The obtained stress, flexural tensile strength, and stress level of the APCP slab were substituted into the fatigue failure formulas previously mentioned to find the number of load applications at which fatigue failure occurred. First of all, in the S-N curve, the stress level is 0.46, which is less than 0.5, so damage due to fatigue does not occur during the number of load repetitions of 10 million. The number of load applications until fatigue failure occurred was analyzed to be 11 million using the Darter formula [60], 1.78 million using the Aas–Jakobsen formula [61], and 7.98 million using the Roesler formula [62].

Gimpo International Airport operates the most airplanes similar to the B737 and A320 airplanes, which showed the greatest strains in the APCP in this experiment, and the number of operations over the past five years is reported to be approximately 260,000 as shown in Table 5 [64]. The rate of increase in the number of flights for these airplanes over 5 years is analyzed to be about 1%, and assuming that this rate of increase is maintained, the number of flights over 30 years is expected to be about 1.7 million. Therefore, even if all of these airplanes operate on the taxiway section where the APCP is constructed, and even if the most conservative fatigue failure prediction formula, the Aas–Jakobsen formula [61], is used, a service life of more than 30 years is secured. In addition, since the stress level value of 0.46 used in this study was obtained using the highest strain during the experiment, if a stress level smaller than this value is applied, the service life will increase further. Moreover, wandering of the gear loads during airplane operation will also increase the service life. Therefore, it is analyzed that the actual service life of the APCP will be well over 30 years. Based on the results of this analysis, it is evaluated that the APCP constructed at Gimpo International Airport by applying new design and construction concepts has secured long-term serviceability.

It should be noted that the fatigue life can be affected by various environmental factors. Unfortunately, however, research on the fatigue failure analysis considering environmental conditions is very limited due to irregular patterns. Recently, the accelerated pavement tests have been performed to evaluate pavement performance considering environmental factors, but the environmental loads were applied using predetermined patterns [65,66,67]. Therefore, the fatigue failure analysis in this study was performed based on existing analysis results applicable to concrete pavement without considering environmental conditions.

## 7. Summary and Conclusions

At Gimpo International Airport in Seoul, Korea, part of the asphalt-paved taxiway was converted to concrete pavement using the precast concrete pavement method involving new design and construction concepts. In this study, the behavior of the APCP was experimentally analyzed and its long-term performance was predicted. During the construction of the APCP, various gauges were installed to measure the behavior of the APCP under environmental and moving airplane loads, and the long-term performance of the APCP was predicted based on the analyzed behavior.

The temperature changes through the depth of the APCP slab were measured and analyzed. The temperature change is largest at the top of the slab, and the temperature change decreases as the slab depth increases. The times at which the maximum and minimum temperatures occur are somewhat delayed as the slab depth increases. The temperature change characteristics of the APCP are basically identical to those generally observed in cast-in-place concrete pavements. Under environmental loads, the daily maximum longitudinal strain is largest at the center of the slab, and the strain decreases somewhat toward the side edges of the slab. When comparing the longitudinal strains with the transverse strains, they are almost similar although the longitudinal strains are very slightly larger. It is also found that the strain change at the bottom of the slab due to the temperature change appears most linearly according to the temperature change at the top of the slab. At night, the central part of the APCP slab is firmly supported by the underlying support layers because the APCP slab curls up, so the horizontal strains in the central part of the slab appear small when the moving airplane loads are applied. During the day, on the other hand, the central part of the APCP slab is not firmly supported by the underlying support layers because the APCP slab curls down, causing the horizontal strains in the central part of the slab to become larger under the moving airplane loads. An analysis was also performed to evaluate the vertical deformation of the APCP under airplane loads, and the results showed that the vertical deformation of the APCP was found to be within the typical range of vertical deformation of concrete pavement. The important findings from this study are provided as follows:

- Based on the overall results of this study, it is concluded that the APCP, which is designed to reduce the slab thickness by placing reinforcing bars in the slab through reinforced concrete structural design, exhibits typical behavior of concrete pavements and can be successfully applied to the airport pavement rehabilitation.
- The vertical displacements at the slab corners are clearly larger than those at the center of the slab edge due to environmental loads. This implies that the APCP slabs are separated from the supporting layer and exhibit curling behavior like typical concrete pavement slabs. It is also noticed that the curling behavior according to the temperature change by slab depth appears most linearly when analyzing based on the temperatures at the top of the slab.
- To analyze the behavior of the APCP under dynamic loads, the dynamic load tests were performed using a heavy weight deflectometer. The strain when the dynamic load acts on the central part of the slab is smaller than when it acts near the slab edge, which implies that the flexural rigidity at the central part of the slab is greater than that near the slab edge. Additionally, the transverse strain is found to be slightly larger than the longitudinal strain at the central part of the slab.
- The dynamic strain responses of the APCP under moving airplane loads were measured during the day and night times separately, and the strains during the day were found to be significantly larger than those at night. These results are caused by the curling phenomenon of the slab.
- The long-term performance of the APCP was evaluated using various fatigue failure formulas. The obtained stress, flexural tensile strength, and stress level of the APCP slab were substituted into the fatigue failure formulas to find the number of load applications at which fatigue failure occurred. Even if the most conservative fatigue failure prediction formula was used, a service life of more than 30 years was secured.

It is noted that further research on the numerical analysis of the APCP could be useful for validation and generalization of the results found in this study.

## Figures and Tables

**Table 1:** Comparison of longitudinal and transverse strains.

Slab	HWD Load Location	Strain Gauge	Transverse Strain	Longitudinal Strain	Strain Ratio	Average Strain Ratio
PC2	H6	S6, S7	49.2	49.8	0.99	1.1
PC3	H8	S10, S11	52.0	42.6	1.22	

**Table 2:** Airplane types and strain measurement times.

Airplane No.	Airplane Type	Airplane Class	Measurement Time	Day or Night
D1	B737	C	15:02	Day
D2	A320	C	15:06	
D3	B737	C	15:08	
D4	B737	C	15:09	
D5	A220	C	15:44	
D6	A320	C	15:45	
D7	B737	C	15:52	
D8	A320	C	15:53	
D9	B747	E	15:56	
D10	B737	C	15:57	
D11	A330	E	16:01	
D12	B737	C	16:02	
N1	B737	C	20:59	Night
N2	B737	C	21:06	
N3	A320	C	21:16	
N4	Lightweight plane	C	21:19	
N5	B737	C	21:39	
N6	A320	C	21:41	

**Table 3:** Maximum strains (με) at gauge locations.

Airplane No.	S1	S2	S3	S4	S5	Max.	Average	Day or Night
D1	67.0	60.9	63.8	37.9	22.8	67.0	60.5	Day
D2	20.0	27.4	43.3	67.7	77.5	77.5		
D3	72.9	63.9	48.3	28.5	16.2	72.9		
D4	63.0	51.4	43.8	29.1	19.3	63.0		
D5	26.8	38.5	46.3	43.1	31.4	46.3		
D6	21.7	33.6	51.5	51.3	62.7	62.7		
D7	36.6	55.2	56.7	54.3	37.8	56.7		
D8	14.0	18.1	21.1	36.7	68.8	68.8		
D9	46.9	49.6	21.0	20.9	20.5	49.6		
D10	39.4	56.0	64.0	48.8	30.9	64.0		
D11	9.7	12.8	15.0	18.5	23.6	23.6		
D12	74.0	60.8	20.1	34.0	25.5	74.0		
N1	41.4	54.1	48.8	43.6	30.1	54.1	48.6	Night
N2	29.8	48.1	39.5	44.8	26.8	48.1		
N3	30.4	46.4	56.7	57.4	46.2	57.4		
N4	30.8	13.2	8.8	6.1	10.1	30.8		
N5	21.8	31.1	46.0	43.3	42.5	46.0		
N6	17.0	24.4	44.3	46.3	55.0	55.0		

**Table 4:** Flexural tensile strength test results of APCP slab.

Item	Teat Method	Specimen No.	Test Result
Flexural tensile strength (28 days)	KS F 2566 [59]	1	5.5 MPa
		2	6.0 MPa
		3	5.8 MPa
		Average	5.77 MPa

**Table 5:** Number of airplanes departing from Gimpo International Airport [64].

Year	B737	B747	B767	B777	A320	A330	Others	Sum
2023	31,098	2	1441	51	18,752	6137	9845	67,326
2022	36,238	0	1184	436	21,754	4053	8202	71,867
2021	37,968	0	1278	361	20,963	3102	5801	69,473
2020	28,120	1	3317	2109	16,381	1888	4985	56,801
2019	33,319	23	5231	6063	14,701	3564	7329	70,230
Sum	166,743	26	12,451	9020	92,551	18,744	36,162	335,697

## Data Availability

Data will be made available on request.
